# Tracheotomy for the Extraction of a Piece of Glass

**Published:** 1853-09

**Authors:** 


*Tracheotomy for the extraction of a piece of glass.*—A boy, aged 8, was brought hastily to the Newcastle Infirmary in a suffocating state, in consequence of a portion of broken glass having got into his wind-pipe during play. He was seen soon after admission, and it was found by the stethoscope, from the distinct whistling sound, most evident a little below the cricoid, that the glass was impacted there, and he was immediately placed under the influence of chloroform. Tracheotomy was then performed, and the trachea slit downwards from the cricoid for about an inch, when a violent convulsive paroxysm of coughing expelled a piece of glass about half an inch long, with irregular edges, and about a quarter of an inch broad. The patient recovered without a bad symptom.—*London Medical Times and Gazette.*

